# Heat stress promotes the accumulation of tomato yellow leaf curl virus in its insect vector by activating heat shock factor

**DOI:** 10.1007/s44297-024-00039-8

**Published:** 2024-12-17

**Authors:** Yu-Meng Wang, Ting Xie, Ya-Zhou He, Wilmer J. Cuellar, Xiao-Wei Wang

**Affiliations:** 1 State Key Laboratory of Rice Biology and Breeding, Ministry of Agriculture Key Lab of Molecular Biology of Crop Pathogens and Insects, Zhejiang Key Laboratory of Biology and Ecological Regulation of Crop Pathogens and Insects, Institute of Insect Sciences, Zhejiang University, Hangzhou, 310058 China; 2 College of Plant Protection, Nanjing Agricultural University, Nanjing, 210095 China; 3 Virology Laboratory, Cassava Program, International Center for Tropical Agriculture (CIAT), Km 17 Recta Cali-Palmira, Cali, 763537 Colombia

**Keywords:** Tomato yellow leaf curl virus, Heat stress, Virus replication, *Bemisia tabaci*, Heat shock factor

## Abstract

High temperature tends to be a contributing factor to the spread of vector-borne viral diseases by enhancing vector competence. However, the underlying mechanisms of the increased virus transmission capacity of vectors under heat stress are still largely unknown. Tomato yellow leaf curl virus (TYLCV) is one of the most devastating plant DNA viruses worldwide and is transmitted exclusively by *Bemisia tabaci*. Here, we identified heat shock factor (HSF) as a key transcription factor that mediates TYLCV accumulation in whitefly vectors under heat stress. Quantitative analysis revealed that the amount of TYLCV DNA in whiteflies gradually increased with increasing temperature (above 38 °C). To determine the underlying mechanism, RNA-seq analysis was performed, which revealed that 1 h of heat stress caused a dramatic increase in heat shock protein (HSP) expression. Moreover, the whitefly HSP transcription factor HSF was shown to specifically interact with the intergenic region of TYLCV via yeast one-hybrid and dual-luciferase analyses. Additionally, the transcriptional activity of HSF gradually increased with increasing heat treatment temperature, indicating a similar pattern of virus accumulation under heat stress. Knocking down *HSF* in whiteflies caused a significant decrease in TYLCV DNA and coat protein. Our results reveal the important role of HSF in mediating virus accumulation in insect vectors under high temperature and provide insights into how environmental factors affect plant virus‒vector interactions.

**Supplementary Information:**

The online version contains supplementary material available at 10.1007/s44297-024-00039-8.

## Introduction

Ambient temperature is a critical factor for the epidemic dynamics of pests and pathogens [1, 2]. Global warming is predicted to expand their distribution to places that used to be unfavorable, which is highly important for both global food security and natural ecosystems [3, 4]. Therefore, changes in environmental temperature may affect vector competence and viral spread. Previous studies have shown that the effect of relatively high temperatures on the transmission ability of vectors is specific to the combination of insect vectors and viruses [5–7]. For example, heat stress can facilitate the transmission of dengue 2 virus via its vector *Aedes aegypti*, and lower temperatures shorten the time for western equine encephalitis virus to circulate in Culex tarsalis [8, 9]. However, the mechanisms by which higher temperatures affect vector–virus interactions are largely unknown.

Tomato yellow leaf curl virus (TYLCV) is one of the most devastating single-stranded DNA viruses and threatens the production and quality of tomatoes worldwide [10]. TYLCV is effectively transmitted by the invasive cryptic species of *Bemisia tabaci*, Middle East Asia Minor 1 (MEAM1), in a circulative manner. As the only structural protein, its coat protein (CP) plays a critical role in interactions with insect vectors [11, 12]. On the basis of previous results, the following reasons explain why MEAM1 whitefly transmitted TYLCV is highly efficient. First, TYLCV could come across *B. tabaci* important transmission barriers with high efficiency, such as midguts (MGs), primary salivary glands (PSGs) for plant‒plant transmission and ovaries for transmission to offspring [13–18]. Furthermore, TYLCV was found to replicate in PSGs, suggesting that TYLCV can be retained in whiteflies throughout their life cycle once it is acquired [19]. More importantly, virus-induced apoptosis can increase TYLCV accumulation and transmission [20, 21]. TYLCV CP can hijack the antiviral role of the JAK/STAT pathway to ensure its survival and transmission [22]. In addition, environmental factors affect virus transmission. Previous studies have shown that heat treatment (40 °C) can facilitate TYLCV transmission and that the occurrence of TYLCV tends to be more severe in tropical and subtropical regions where temperatures are relatively high [23]. Nonetheless, little is known about the mechanisms underlying the temperature-induced changes in whiteflies that affect TYLCV transmission.

The heat shock response is first observed as increased transcriptional activity of particular *loci* on polytenic chromosomes in *Drosophila melanogaster* at 37 °C [24] and then appears to be a common phenomenon both in homothermic and poikilothermic organisms [25]. When exposed to high temperature, insects respond to changes in their physiology and behavior to adapt to new conditions, involving neurons and neurotransmitters to detect environmental signals, heat shock proteins (HSPs) to prevent cell damage, and hormones to regulate development and behavior [26]. The best studied stress proteins are HSPs, which often work in networks to maintain cell homeostasis through interactions with substrate proteins [27]. HSP transcription induced by elevated temperature relies on the activation of a transcription factor, heat shock factor (HSF), which may involve trimerization and phosphorylation, increasing the ability of HSPs to bind DNA [28]. While activated, HSF induces the transcription of genes containing conserved heat-shock elements (usually containing an nGAAn motif) in their promoters [29]. In MED *B. tabaci*, BtHSF was shown to be induced by both heat stress and cold stress, and the knockdown of HSF decreased temperature stress tolerance [30]. However, whether and how HSF mediate the effects of temperature on the interaction between whiteflies and the viruses they transmit are still unclear.

As an important environmental factor, the possible mechanism by which temperature affects virus transmission by insect vectors has received increasing attention [7, 31]. However, few studies have clarified the underlying mechanisms involved. In this study, we found that BtHSF could directly interact with the intergenic region of TYLCV to promote its downstream gene expression and then facilitate TYLCV accumulation in whiteflies. Our findings reveal a novel mechanism by which environmental temperature affects virus accumulation in insect vectors and provide insights into the relationship between high temperature and virus epidemics.

## Materials and methods

### Insects and virus-infected plants

A population of MEAM1 of the *B. tabaci* cryptic species complex was used in this study. Whiteflies were reared on cotton plants (*Gossypium hirsutum* L. cv. Zhemian 1793) in insect-proof cages at 27 °C under a photoperiod of 14:10 h (light/dark) and a relative humidity of 60%. The purity of the population was monitored every three generations by amplifying and sequencing the mitochondrial cytochrome oxidase I gene (GenBank accession no. GQ332577, using the primer COI shown in Table S2), which has been widely used to differentiate *B. tabaci* cryptic species [32]. Infectious clones of TYLCV isolate SH2 (GenBank accession no. AM282874) were agroinoculated into 3–4 true leaf stage tomato plants (*Solanum lycopersicum* cv. Hezuo 903), and the plants were used for virus acquisition approximately 4 weeks post virus inoculation. All the plants were grown in insect-proof greenhouses at a controlled temperature of 25 ± 3 °C with natural lighting.

### Heat treatment

For virus accumulation analysis, newly emerged whiteflies were first given a 6 h acquisition access period (AAP) on TYLCV-infected plants and then kept at different temperatures (27, 34, 36, 38, 40, 42 °C) for 1 h. Groups of 20 whiteflies were collected for DNA extraction. For gene transcription analysis, newly emerged whiteflies were incubated at different temperatures (27, 34, 36, 38 °C) for 1 h, and groups of 40 whiteflies were collected for RNA extraction.

### Quantification of viruses and analysis of gene transcription

For viral DNA quantification, groups of 20 whiteflies each were ground in 40 μL of ice-cold lysis buffer (50 mM Tris–HCl (pH 8.4), 0.2% gelatin, 0.45% Tween 20, 0.45% Nonidet P-40, and 60 mg/l proteinase K), incubated at 65 °C for 2 h, and then boiled in water for 10 min. The supernatants were subjected to quantitative PCR (qPCR). For analysis of gene transcription, total RNA was isolated from groups of 40 whitefly whole bodies, groups of 100 MGs or PSGs each using TRIzol reagent (Ambion), and then cDNAs were generated via the PrimeScript RT reagent kit with gDNA Eraser (TaKaRa). MGs were dissected from the abdomen in prechilled PBS buffer, and PSGs were dissected from the prothorax. Thereafter, they were washed twice in PBS to remove contamination from the hemolymph. qPCR was performed via a CFX Connect Real-Time PCR System (Bio-Rad) with SYBR Premix Ex TaqTM II (TaKaRa), and the primers (qTYLCV, qHSPs, qBtHSF and q-β-actin) used are shown in Table S2. For each reaction, 0.8 μL of each primer (10 mM), 6.4 μL of nuclease-free water, 2 μL of sample and 10 μL of SYBR Premix Ex Taq were added to a total volume of 20 μL. The qPCR protocol was 95 °C for 30 s, followed by 40 cycles at 95 °C for 5 s and 60 °C for 30 s. A negative control (using nuclease-free water instead of sample) was included throughout the experiments to detect contamination and to determine the degree of dimer formation.

### RNA-seq analysis

For RNA-seq analysis, newly emerged whiteflies were treated at 38 °C for 1 h, and the control group was maintained at 27 °C (a rearing temperature for whiteflies). After heat shock, groups of 80 whiteflies were collected and immediately frozen in liquid nitrogen. Three biological replicates were used in this study. RNA-seq was performed by LC-Bio Technology Co. Ltd., Hangzhou, China, with a next-generation sequencing platform (Illumina NovaseqTM 6000). The raw data were submitted to NCBI (NCBI, Bethesda, USA) with the accession number PRJNA1141957. The RNA-seq reads were aligned to the *B. tabaci* reference genome (genome assembly ASM185493v1) via TopHat (v2.0.13) after low-quality reads were filtered out. Differentially expressed genes were evaluated via the DESeq R package. Genes with q < 0.05 (adjusted p value) and a log_2_ ratio > 1 were considered differentially expressed. Web GO functional annotation plots and the KEGG pathway database were used to investigate the functions of the DEGs. Bioinformatic analysis was performed via the OmicStudio tools at https://www.omicstudio.cn/tool.

### Yeast one-hybrid (Y1H) assay

The Y1H assay was performed via the Matchmaker Gold Yeast One-Hybrid Library Screening System and Yeastmaker Yeast Transformation System (Clontech) as described in the manufacturer’s instructions. A cDNA library of *B. tabaci* was constructed in the prey plasmid SfiI-digested pGADT7 via the SMART cDNA library construction kit (Clontech). The bait vector was constructed by fusing three tandem copies of the TYLCV intergenic region (5’-TTTATTGATTTGATTTTTGAATTTTGAATTTTGAATTGCAATGTACTTTTACAAAATTACCAAATAGCCATTAGGTGTCCAGGTATAAGTAAGACACCGATACACCGATTGCCATAGAGCTTTGAGGGACACCAATTCATTTCAAC-3’) into pAbAi with *SacI* and *SalI*. To confirm that the plasmid was integrated correctly, we used colony PCR analysis with Matchmaker Insert Check PCR Mix (Clontech). After screening different concentrations of aureobasidin A (AbA) to suppress the basal expression of bait constructs, we detected no background growth of yeast when the AbA concentration was 200 ng/ml. The cDNA library was then transformed into yeast containing integrated parts of the intergenic region. Plasmids from the positive clones were recovered, transformed into the *Escherichia coli* strain DH5α and sequenced thereafter. To further confirm the interaction, we cotransformed prey plasmids into bait yeast and spotted them on selective media (-Leu + AbA).

### Cell culture and plasmid transfection

*Drosophila* Schneider S2 cells were maintained at 27 °C in *Drosophila* serum-free medium supplemented with 10% heat-inactivated fetal bovine serum (Gibco). pAc5.1/V5-HisB (Invitrogen) was used for BtHSF expression. The wild-type and mutated sequences of the TYLCV intergenic region were chemically synthesized and subcloned into the reporter vector pGL3-Basic (Promega) via the primers pAc5.1-BtHSF and TYLCV-P M1, M2, WT, as shown in Table S2. The constructs were confirmed by sequencing. Twelve hours before transfection, S2 cells were seeded in a 24-well plate at 2 × 10^5^ cells per well. Transfections with expression plasmids, reporter plasmids and Renilla luciferase plasmids were conducted via Lipofectamine 3000 (Invitrogen). At 72 h posttransfection, the cells were harvested and processed with a Dual-Luciferase Reporter Assay Kit (Vazyme) according to the manufacturer's protocol. The luciferase activities were measured via a FlexStation-3 microplate reader (Molecular Devices). The activity of firefly luciferase was normalized to that of Renilla luciferase. All the experiments were carried out in triplicate.

### Gene silencing by oral ingestion of double-stranded RNA (dsRNA)

DsRNA was synthesized via the T7 High Yield RNA Transcription Kit (Vazyme) following the manufacturer’s instructions. Briefly, the DNA template for dsRNA synthesis was amplified with primers containing the T7 RNA polymerase promoter at both ends. The primers used for dsRNA-GFP *(green fluorescent protein)* and dsRNA-BtHSF are shown in Table S2, and the purified DNA template was then used to synthesize dsRNAs. The synthesized dsRNA was subsequently purified via phenol‒chloroform precipitation and resuspended in nuclease-free water. The concentration of dsRNA was determined with a NanoDrop 2000 (Thermo Fisher Scientific). Moreover, the quality and size of the dsRNAs were further verified via electrophoresis in a 2% agarose gel. For the oral ingestion of dsRNA, the feeding chambers were made with glass tubes (diameter: 1.5 cm, length: 10 cm). One end of each tube was covered with two layers of parafilm that were filled with a 15% (wt/vol) sucrose diet containing dsRNA at a concentration of 250 ng/μl. Approximately 100 adult whiteflies were placed in each feeding chamber and allowed to feed for 48 h. For virus accumulation analysis, newly emerged whiteflies were first given a 6 h AAP on TYLCV-infected plants and then collected for dsRNA feeding. Groups of 20 whiteflies were subsequently collected for DNA extraction, groups of 40 whiteflies were collected for RNA extraction, and groups of 100 whiteflies were collected for protein extraction.

### Western blot

To detect TYLCV CP in whiteflies, samples of 200 dsRNA-treated whiteflies each described in section 2.7 were prepared via radioimmunoprecipitation assay buffer with proteinase inhibitors. Protein samples were separated using 4-20% sodium dodecyl sulfate‒polyacrylamide gel electrophoresis and then transferred onto a PVDF membrane. The membrane was blocked with 5% milk in TBST (10 mM Tris HCl, 150 mM sodium chloride, 0.05% Tween 20, pH 7.5) and then incubated with antibodies against TYLCV CP (kindly provided by Professor Jian-Xiang Wu, Zhejiang University) or beta-actin (EARTHOX Life Sciences). After incubation with horseradish peroxidase (HRP)-conjugated secondary antibodies, the blots were visualized with an enhanced chemiluminescence (ECL) Plus detection system (Bio-Rad).

### Immunofluorescence assay

To visualize TYLCV in whitefly tissues, MGs and PSGs were dissected from female whiteflies. The samples were fixed in 4% paraformaldehyde for 1 h at room temperature and then washed three times in TBS containing 0.1% Triton X-100. The samples were subsequently blocked in TBS containing 1% BSA for 2 h at room temperature, followed by incubation with anti-TYLCV CP monoclonal antibody (1:500) overnight at 4 °C and then with goat anti-mouse (1:500) secondary antibody labeled with Dylight 549 (MultiSciences Biotec) in TBST containing 1% BSA for 1 h at room temperature after extensive washing. After extensive washing in TBST, the MGs and PSGs were mounted in fluoroshield mounting medium with DAPI (Abcam) and imaged on a Zeiss LSM710 confocal microscope (Zeiss).

### Statistical analysis

The relative abundance of viral DNA and relative level of gene expression were calculated via the 2^−Δ Ct^ method and normalized to the level of the *B. tabaci β-actin* gene. The data are presented as the means ± SEMs of three independent biological replicates, unless otherwise noted. One-way ANOVA followed by the least significant difference test was used for multiple comparisons. Independent-sample t tests were performed to determine significant differences (*p* < 0.05) between treatments. All analyses were performed via SPSS 13.0.

## Results

### TYLCV rapidly accumulates in whiteflies under heat stress

**Fig. 1:**
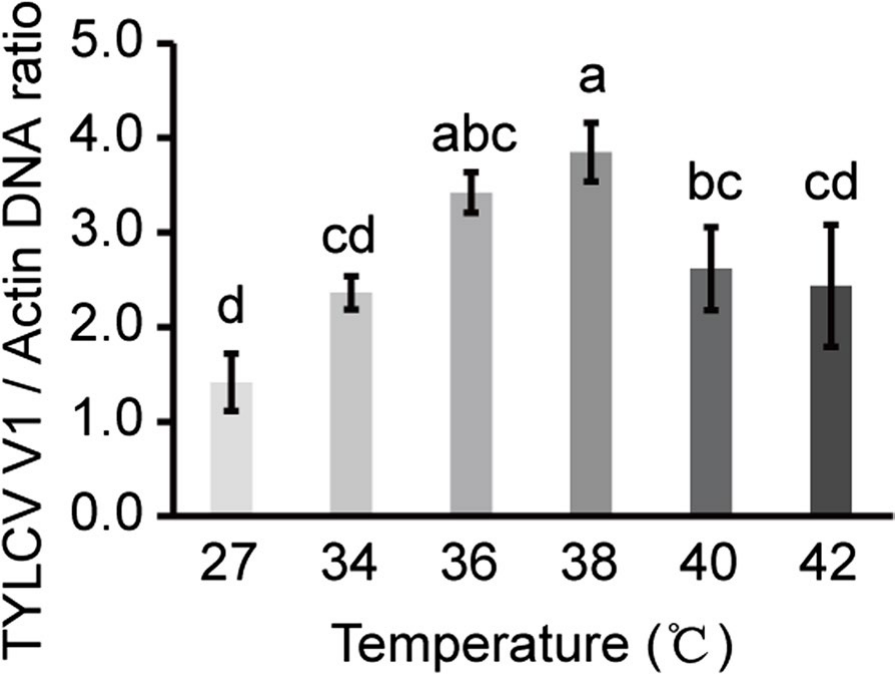
Effect of heat stress on TYLCV accumulation in whiteflies. Quantitative analysis of viral DNA in whiteflies subjected to heat stress for 1 h after a 6-h AAP on TYLCV-infected tomato plants. The data are presented as the means ± SEMs of three independent experiments and were analyzed statistically via one-way ANOVA and the LSD test (*p* < 0.05)

To investigate the effect of heat stress on TYLCV replication in whiteflies, newly emerged whiteflies were subjected to heat stress for 1 h after a 6-h AAP on TYLCV-infected tomato plants. Quantitative analysis revealed that the abundance of TYLCV DNA in whiteflies gradually increased with increasing temperature. When whiteflies were treated at 38 °C for 1 h, the amount of virus in whiteflies peaked and then decreased with increasing temperature. Interestingly, while the amount of viral DNA in *B. tabaci* treated at 40 °C or 42 °C for 1 h was greater than that in the control group (27 °C), it was significantly lower than that in the 38 °C treatment group (Fig. 1). These results demonstrate that heat stress below 38 °C facilitates TYLCV accumulation in whiteflies.

### Transcriptional response of whiteflies to heat stress

**Fig. 2:**
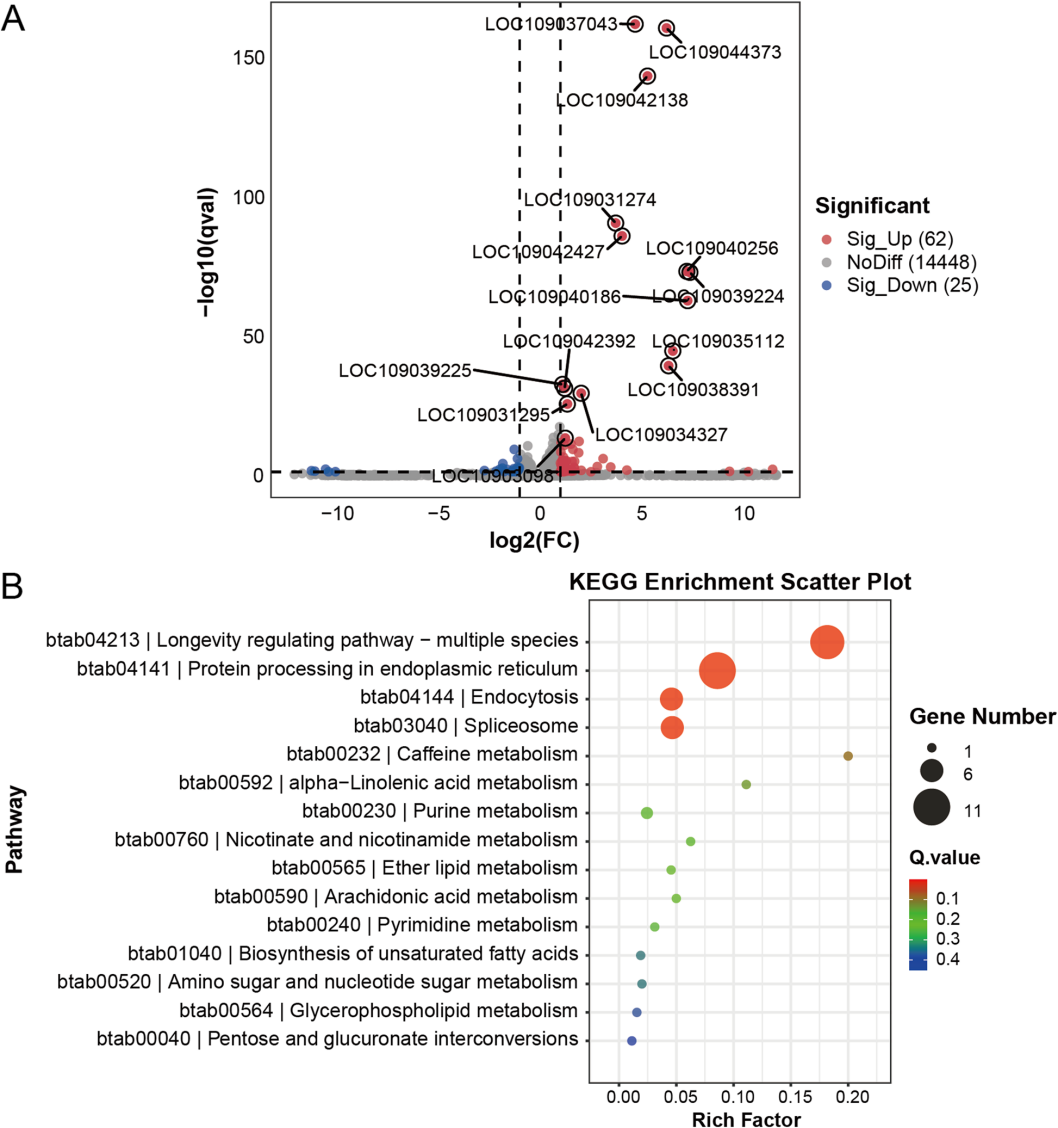
RNA-seq data analysis. **A** Volcano plot of downregulated (blue), upregulated (red) and nonsignificant (gray) genes at a log_2_fold change of 1 and an adjusted p value of < 0.05. **B** KEGG enrichment analysis. The rich factor is the ratio of the number of differentially expressed genes annotated in this pathway term to the total number of genes annotated. A greater rich factor means greater intensiveness. The Q value ranges from 0⁓1, and a lower Q value indicates greater intensiveness

To gain further insight into the molecular mechanisms underlying virus accumulation in whiteflies triggered by heat stress, we used RNA-Seq to investigate the transcriptional response of whiteflies to heat treatment. We found 62 upregulated and 25 downregulated genes in heat-treated whiteflies compared with those in the control group (Fig. 2A). KEGG analysis revealed that the longevity regulating pathway, protein processing in the endoplasmic reticulum, the spliceosome and endocytosis were significantly enriched (Fig. 2B). The DEGs enriched in these pathways were heat shock proteins and lethal (2) essential for life-like proteins (Table S1). Consistent with the findings in other insects, these genes were upregulated in response to heat stress. Moreover, we also identified 4 upregulated genes and 3 downregulated genes involved in transcription or protein folding (Table S1), which may be involved in viral gene transcription and the synthesis of viral proteins in whiteflies.

### HSF directly binds to the intergenic region of TYLCV

**Fig. 3:**
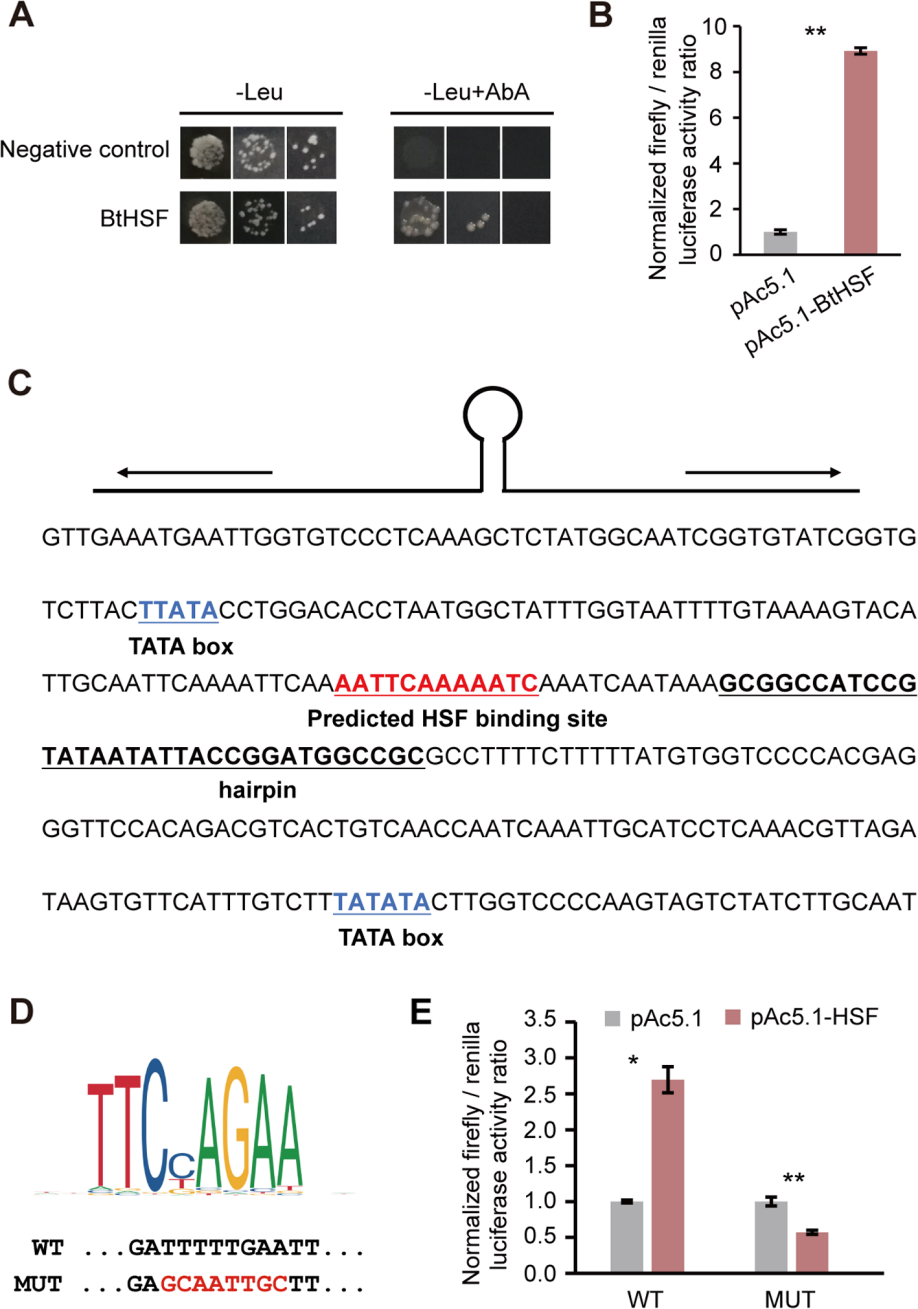
Identification of the interactions between the TYLCV intergenic region and whitefly HSF. **A** Verification of the interaction between whitefly HSF and the TYLCV intergenic region via a Y1H assay. The concentration of aureobasidin A (AbA) used in the selective medium (-Leu + AbA) was 200 ng/ml. Yeast growth on selective media (-Leu + AbA) was recorded on day 3 as an indicator of protein‒DNA interactions. The empty pGAD vector served as a negative control. **B** Luciferase reporter assays after cotransfection of the expression vector pAc5.1-BtHSF and the reporter vector pGL3-basic with the TYLCV intergenic region into S2 cells. **C** The virion-sense DNA sequence of the TYLCV intergenic region, which corresponds to positions 2616 to 147 in the TYLCV genome, is shown. The hairpin structure, TATA box and predicted HSF binding site are underlined. **D** The HSF matrix was downloaded from JASPAR (http://jaspar.genereg.net/), and the matrix ID was MA1458.1. Putative HSF-binding elements in the TYLCV intergenic region and the mutated sequences used for the experiments are shown. **E** Luciferase reporter assays after the cotransfection of the expression vector pAc5.1-BtHSF and the reporter vector pGL3-basic with the TYLCV intergenic region (WT) or mutant sequence (MUT) into S2 cells. **B** and **E** Treatments with an empty expression vector served as controls. Firefly luciferase activity was normalized to Renilla luciferase activity. The data are presented as the means ± SEMs of three or four independent experiments and were analyzed statistically via independent-samples t tests (*, *p* < 0.05; **, *p* < 0.01)

To identify the whitefly protein involved in TYLCV replication, we used the TYLCV intergenic region as bait to screen the cDNA library of whiteflies in a yeast one-hybrid system. The transcription factor HSF, which functions as a regulator of HSP expression [28], was isolated. Bait yeast transformants with the pGADT7-HSF plasmid were able to grow on selective media, whereas yeast transformants carrying the control construct were unable to do so (Fig. 3A). After a BLAST search of the *B. tabaci* genome, the full-length open reading frame (ORF) of BtHSF was identified and cloned (GenBank accession no. XM_019050677.1). Its 2283-bp nucleotide gene encodes a 760-amino acid protein with a predicted molecular mass of 84.1 kDa. Domain architecture analysis of BtHSF revealed the presence of a conserved functional domain, the HSF_DNA-binding domain, at amino acid residues 14–144 (Fig. S1). qRT‒PCR analysis revealed that *BtHSF* was expressed both in MGs and PSGs (Fig. S2), which are critical tissues for TYLCV replication and transmission [12, 19]. To investigate the regulatory effect of BtHSF on TYLCV genes, the promoter was cloned and inserted into the luciferase reporter vector pGL3basic and cotransfected with the expression vector pAc5.1 into the cell culture system. Compared with the control (transfected with the empty expression vector), BtHSF overexpression resulted in a more than eightfold increase in luciferase activity when the intergenic region was cloned and inserted into the reporter vector (Fig. 3B).

To pinpoint the specific binding sites of BtHSF, we scanned the TYLCV intergenic region via the HSF matrix in the *Drosophila* database of JASPAR and identified a putative binding site, TTTTTGAA, which is located in the complementary sense of the TYLCV intergenic region (Fig. 3C). To demonstrate the specificity of the interaction between BtHSF and this binding site, we mutated TTTTTGAA to GCAATTGC (MUT) (Fig. 3D). The dual-luciferase assay revealed that mutation of this binding site caused a decrease but not an increase in luciferase activity when BtHSF was overexpressed (Fig. 3E), demonstrating the specific interaction between BtHSF and the predicted binding site in the TYLCV intergenic region. Taken together, these data confirmed that the whitefly HSF specifically binds to the TYLCV intergenic region and can activate the transcription of its downstream genes.

### Heat stress enhances the transcriptional activity of BtHSF

**Fig. 4:**
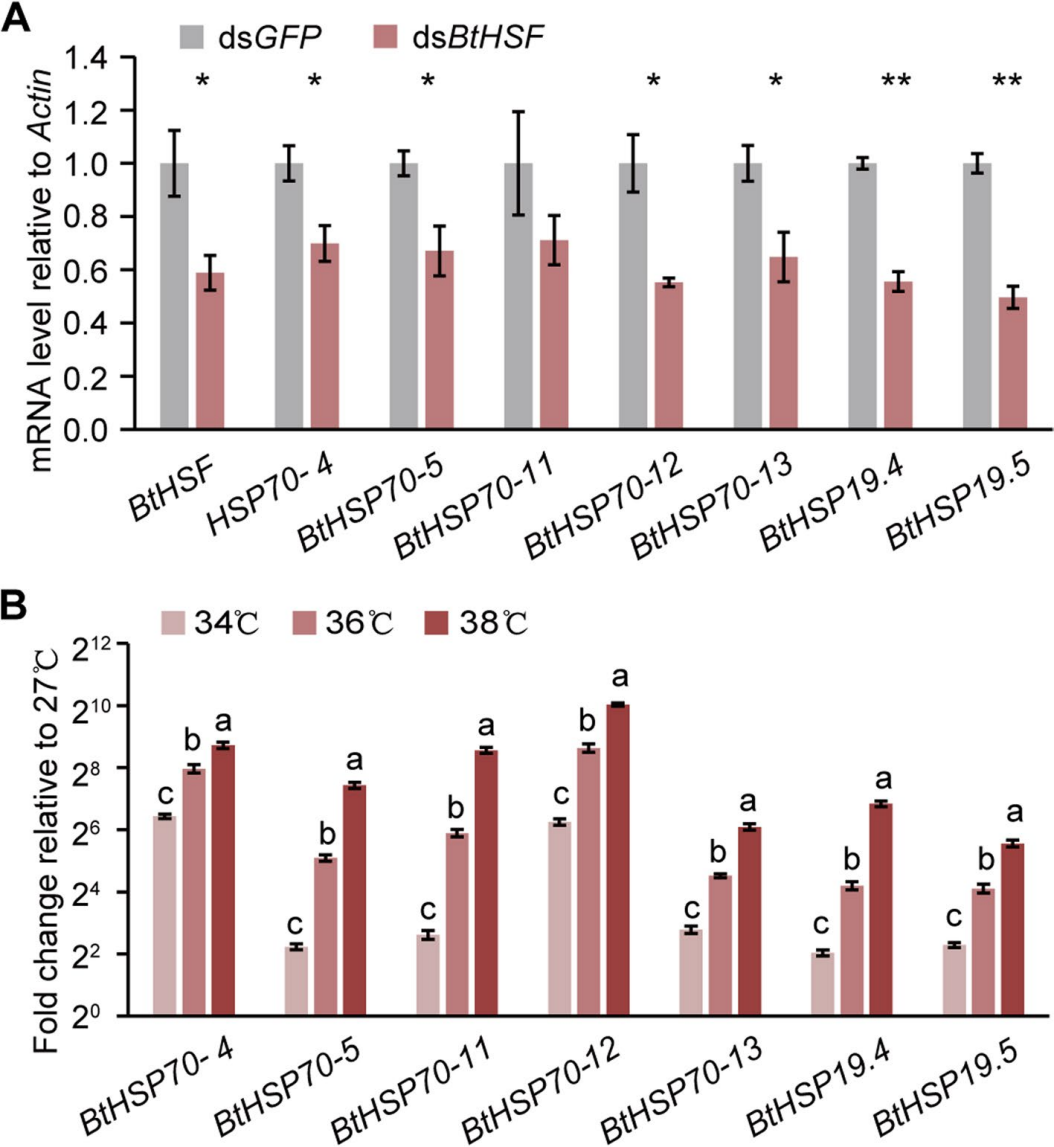
Effect of heat stress on HSF transcriptional activity. **A** Quantitative analysis of the mRNA levels of *BtHSF*, *BtHSP70-4*, *BtHSP70-5*, *BtHSP70-11*, *BtHSP70-12*, *BtHSP70-13*, *BtHSP19.4* and *BtHSP19.5* after dsRNA treatment. The data are presented as the means ± SEMs of three independent experiments and were analyzed statistically via independent-samples t tests (*, *p* < 0.05; **, *p* < 0.01). **B** Fold change in HSP mRNA levels after heat treatment compared with those at the control temperature (27 °C). Mean ± SEM from three independent experiments, *p* < 0.05 (one-way ANOVA, LSD test)

On the basis of the above results, we speculated that BtHSF may play a role in mediating virus accumulation under heat stress. To test this hypothesis, we first verified the relationship between BtHSF activity and environmental temperature. As reported in other species, HSF transcriptional activity is closely related to the HSP expression level [29]. We thus examined the transcript levels of HSPs after knocking down the expression of *BtHSF* in non-viruliferous whiteflies. Compared with those in the ds*GFP*-treated group, the transcript levels of *BtHSF* and most of the *HSPs* tested (*BtHSP70-4*, *BtHSP70-5*, *BtHSP70-12*, *BtHSP70-13*, *BtHSP19.4* and *BtHSP19.5*) were significantly lower in the ds*BtHSF*-treated whiteflies (Fig. 4A), confirming that BtHSF controls the transcription of HSPs in whiteflies. Furthermore, the transcriptional patterns of HSPs under different temperature treatments were examined. After normalization to the expression level of whiteflies under the control temperature (27 °C), we found that heat stress (above 34 °C) caused at least a 47-fold increase in the number of HSPs, which was essentially the same as the transcriptome results. Interestingly, the expression of these genes gradually increased with increasing heat treatment temperature (Fig. 4B), indicating a similar pattern of virus accumulation under heat stress. Taken together, our results demonstrated that the transcriptional activity of whitefly HSF increased with increasing environmental temperature.

### Whitefly HSF promotes TYLCV accumulation in whiteflies

**Fig. 5:**
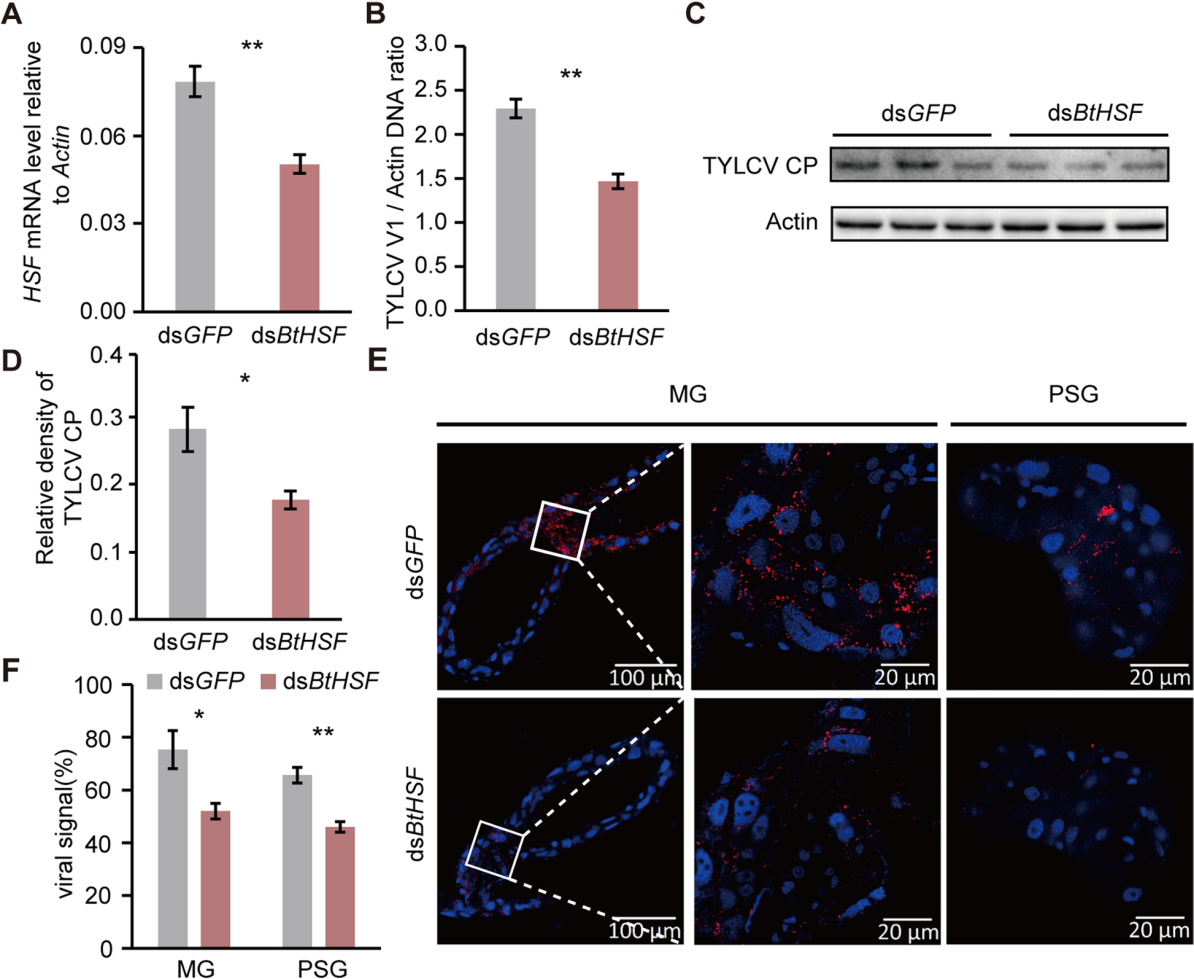
BtHSF promotes TYLCV accumulation in whiteflies. **A** Quantitative analysis of *BtHSF* mRNA levels after feeding with dsRNAs. **B**-**F** Effects of knocking-down *BtHSF* expression on TYLCV replication in whiteflies. Quantitative analysis of TYLCV DNA (**B**) and immunoblot analysis of TYLCV CP (**C**) in whiteflies fed dsRNAs after a 6 h AAP on TYLCV-infected tomato plants. **D** The relative densities of TYLCV CP were normalized to those of Actin. **E** Immunofluorescence staining of TYLCV CP in the midgut and primary salivary glands of dsRNA-treated whiteflies. TYLCV CP was detected via a mouse anti-CP monoclonal antibody and goat anti-mouse IgG labeled with Dylight 549 (red) secondary antibody. The cell nuclei were stained with DAPI (blue). Images are representative of 24 whiteflies analyzed for each treatment. **F** Proportion of TYLCV-positive MG and PSG. The data are presented as the means ± SEMs of three independent experiments and were analyzed statistically via independent simple t-test (*, *p* < 0.05; **, *p* < 0.01)

To determine the role of BtHSF in TYLCV accumulation in whiteflies, we first examined the expression pattern of *BtHSF* in response to TYLCV infection. The results showed that the mRNA levels of *BtHSF* were significantly upregulated in viruliferous whiteflies compared with nonviruliferous whiteflies (Fig. S3), indicating that TYLCV infection induces the expression of *BtHSF*. We subsequently knocked down the expression of *BtHSF* via RNAi. After 6 h of acquisition on TYLCV-infected tomato plants, whiteflies were fed ds*BtHSF* or ds*GFP* for 48 h. Compared with those in the control group, the expression levels of BtHSF were 36% lower in the ds*BtHSF*-treated whiteflies (Fig. 5A). qPCR analysis revealed that the abundance of TYLCV DNA was significantly lower in ds*BtHSF*-treated whiteflies than in the control whiteflies after 48 h of dsRNA treatment (Fig. 5B), indicating that BtHSF had a positive effect on TYLCV accumulation in whiteflies. Compared with that in the control whiteflies, the viral CP level in the ds*BtHSF*-treated whiteflies significantly decreased, as determined by western blotting (Fig. 5C and D). Immunofluorescence assays using the anti-TYLCV CP antibody further revealed that the proportions of TYLCV-positive MGs and PSGs in ds*GFP*-treated whiteflies were 75% and 66%, whereas only 52% and 46% of virus-positive MGs and PSGs were found in the ds*BtHSF*-treated controls (Fig. 5E and F). Together, these data indicate that BtHSF promotes TYLCV replication in whiteflies.

## Discussion

High temperature usually contributes to epidemics and the spread of vector-transmitted plant viruses [2, 3]. One possible reason is that high temperature could promote the growth and development of insect vectors and broaden their distribution to places that are unfavorable for survival [26]. Another possibility is that virus replication may be accelerated by heat stress in plants [33]. In addition, high temperature can affect the interaction between plant viruses and their insect vectors, further regulating virus spread [34]. However, the possible role of heat stress in plant virus‒vector interactions and the underlying mechanisms are still unknown. Here, we used TYLCV and its vector *B. tabaci* as a system to answer this question. Our results demonstrated that heat treatment (below 38 °C) could increase TYLCV accumulation in whiteflies, which was possibly mediated by the temperature-related transcription factor HSF. The whitefly HSF can specifically bind to the intergenic region of TYLCV and positively regulate downstream gene transcription. Therefore, this study sheds light on the effects of environmental temperature on virus‒vector interactions and their underlying mechanisms, which may help explain the rapid spread of vector-borne viruses at high temperatures.

HSF is an important transcription factor that responds to heat stress in eukaryotes and interacts with cofactors to activate or inhibit downstream gene expression by binding to heat shock elements located in their promoters [35]. The prediction of HSF binding sites by JASPR revealed a heat shock element in the intergenic region of TYLCV. Yeast one-hybrid and luciferase analyses demonstrated the specific interaction between TYLCV and HSF, indicating a possible role of HSF in regulating viral gene transcription. To verify this speculation, RNAi experiments were performed, which revealed that the knockdown of BtHSF caused a significant decrease in TYLCV DNA and CP levels. More importantly, the ratio of virus-positive MGs and PSGs labeled with the TYLCV CP antibody obviously decreased after RNAi treatment, suggesting that BtHSF facilitates virus accumulation in these critical whitefly barriers and may further affect virus transmission. In this study, we clarified the role of HSF in promoting TYLCV accumulation in MEAM1 cryptic species of whitefly. For another invasive species, MED, which has a broader distribution in China, whether MED HSF have similar functions and influence virus accumulation is worth further investigation. Since the DNA-binding domains of MEAM1 and MED HSFs are the same, we speculate that MED HSF might also be able to bind the intergenic region of TYLCV. Interestingly, similar mechanisms were also found in animal virus‒host interactions [36]. Studies have shown that HSF contributes to the replication of animal viruses, including dengue virus and vaccinia virus, in their hosts [37, 38]. Moreover, infection with vaccinia virus can activate HSF to regulate the expression of its downstream genes [37]. In addition, HSF directly binds to the LTR promoter of human immunodeficiency virus-1 and the BamHI-Q promoter of Epstein‒Barr virus, promoting virus transcription and replication [39, 40]. Therefore, the ability of HSF to promote virus replication is likely conserved in hosts and vectors. Furthermore, considering the conserved function of HSF in both plants and animals, HSF may also play an important role in plant virus replication.

Our results revealed that heat stress accelerated virus accumulation in whiteflies and that HSF is a key transcription factor involved in this process. This finding also indicates that the increase in virus accumulation in whiteflies under high temperature may be attributed to increased virus transcription after heat treatment. In addition to the transcription factors of the HSPs studied here, RNA-seq analysis revealed that several genes containing transcriptional activity, such as activating transcription factor 3 (ATF3), zinc finger BED domain-containing protein 1-like (ZBED1) and dorsal root ganglia homeobox factor (DRGX), were differentially expressed after heat treatment. ATF3 is a member of the ATF/CREB family of transcription factors that binds to a cAMP response element with the sequence 5’-TGACGTCA in the form of homodimers or heterodimers with other members of the ATF/CREB family and positively regulates various cellular pathways [41, 42]. Several studies have shown that ATF3 can directly regulate viral gene expression [43, 44]. The upregulation of ATF3 after heat stress treatment indicates a potential way for ATF3 to affect TYLCV accumulation in whiteflies. ZBED1 is known as the DNA replication-related element binding factor DREF, and its target genes play roles in various biological processes, including DNA replication, cell cycle regulation, protein synthesis, and protein degradation [45]. The DRGX is a paired-like homeodomain transcription factor necessary for the development of primary sensory neurons in mammals [46]. However, we found that ZBED1 and DRGX were downregulated after heat shock treatment, suggesting that they may indirectly influence virus accumulation by regulating the expression of antiviral genes. Even so, whether these genes are involved in heat-induced virus accumulation needs further investigation.

The effect of high temperature on virus accumulation is not only to promote virus replication but also to influence the immune system of the vector [25]. An increasing number of reports have shown that the temperature-dependent immune response may be due to the promotion of the synthesis of HSPs, which further act as danger signals in immunity [47]. Like heat stress, infection by pathogens also triggers the expression of HSPs [48, 49]. Moreover, the upregulation of the transcription of HSPs affects their resistance to pathogens [50]. Furthermore, similar results have been obtained for TYLCV-whitefly interactions. While TYLCV infection induces the expression of whitefly HSP70, silencing HSP70 facilitates TYLCV accumulation in *B. tabaci* [51]. Notably, our results revealed a decrease in TYLCV abundance at temperatures above 38 °C. This might be the result of enhanced insect immunity triggered by large amounts of virus accumulated in whiteflies or high quantities of HSPs synthesized under high-temperature conditions.

## Supplementary Information

Supplementary Material 1.

## Data Availability

All data generated or analyzed during this study are included in this published article [and its supplementary information files]. Illumina sequencing data have been submitted to the National Center for Biotechnology Information (NCBI) Sequence Read Archive (SRA) under Bio-Project PRJNA1141957.

## References

[1] Skendi S, Zovko M, Ivkovi IP, Lei V, Lemi D. The impact of climate change on agricultural insect pests. Insects. 2021;12:440. 34066138 10.3390/insects12050440 PMC8150874

[2] Velásquez AC, Castroverde CDM, He SY. Plant–pathogen warfare under changing climate conditions. Curr Biol. 2018;28:R619–34. 29787730 10.1016/j.cub.2018.03.054 PMC5967643

[3] Jones RAC. Future scenarios for plant virus pathogens as climate change progresses. Adv Virus Res. 2016;95:87–147. 27112281 10.1016/bs.aivir.2016.02.004

[4] Deutsch CA, Tewksbury JJ, Tigchelaar M, Battisti DS, Merrill SC, Huey RB, Naylor RL. Increase in crop losses to insect pests in a warming climate. Science. 2018;361:916–9. 30166490 10.1126/science.aat3466

[5] Reinhold JM, Lazzari CR, Lahondère C. Effects of the environmental temperature on *Aedes aegypti* and *Aedes albopictus* mosquitoes: a review. Insects. 2018;9:158. 30404142 10.3390/insects9040158 PMC6316560

[6] Ciota AT, Keyel AC. The role of temperature in transmission of zoonotic arboviruses. Viruses. 2019;11:1013. 31683823 10.3390/v11111013 PMC6893470

[7] Bellone R, Failloux AB. The role of temperature in shaping mosquito-borne viruses transmission. Front Microbiol. 2020;11:584846. 33101259 10.3389/fmicb.2020.584846 PMC7545027

[8] Watts DM, Burke DS, Harrison BA, Whitmire RE, Nisalak A. Effect of temperature on the vector efficiency of *Aedes aegypti* for dengue 2 virus. Am J Trop Med Hyg. 1987;36:143–52. 3812879 10.4269/ajtmh.1987.36.143

[9] Kramer LD, Hardy JL, Presser SB. Effect of temperature of extrinsic incubation on the vector competence of *Culex tarsalis* for western equine encephalomyelitis virus. Am J Trop Med Hyg. 1983;32:1130–9. 6625067 10.4269/ajtmh.1983.32.1130

[10] Prasad A, Sharma N, Hari-Gowthem G, Muthamilarasan M, Prasad M. Tomato yellow leaf curl virus: impact, challenges, and management. Trends Plant Sci. 2020;25:897–911. 32371058 10.1016/j.tplants.2020.03.015

[11] Czosnek H, Hariton-Shalev A, Sobol I, Gorovits R, Ghanim M. The incredible journey of begomoviruses in their whitefly vector. Viruses. 2017;9:273. 28946649 10.3390/v9100273 PMC5691625

[12] Wang XW, Blanc S. Insect transmission of plant single-stranded DNA viruses. Annu Rev Entomol. 2020;66:389–405. 32931313 10.1146/annurev-ento-060920-094531

[13] He YZ, Wang YM, Yin TY, Cuellar WJ, Liu SS, Wang XW. Gut-expressed vitellogenin facilitates the movement of a plant virus across the midgut wall in its insect vector. mSystems. 2021;6:e0058121. 34100642 10.1128/mSystems.00581-21 PMC8269243

[14] Zhao J, Chi Y, Zhang XJ, Wang XW, Liu SS. Implication of whitefly vesicle associated membrane protein-associated protein B in the transmission of tomato yellow leaf curl virus. Virology. 2019;535:210–7. 31319278 10.1016/j.virol.2019.07.007

[15] Xia WQ, Liang Y, Chi Y, Pan LL, Zhao J, Liu SS, Wang XW. Intracellular trafficking of begomoviruses in the midgut cells of their insect vector. PLoS Pathog. 2018;14:e1006866. 29370296 10.1371/journal.ppat.1006866 PMC5800681

[16] Zhao J, Lei T, Zhang XJ, Yin TY, Wang XW, Liu SS. A vector whitefly endocytic receptor facilitates the entry of begomoviruses into its midgut cells via binding to virion capsid proteins. PLoS Pathog. 2020;16:e1009053. 33270808 10.1371/journal.ppat.1009053 PMC7714154

[17] Wei J, He YZ, Guo Q, Guo T, Liu YQ, Zhou XP, Liu SS, Wang XW. Vector development and vitellogenin determine the transovarial transmission of begomoviruses. Proc Natl Acad Sci U S A. 2017;114:6746–51. 28607073 10.1073/pnas.1701720114 PMC5495249

[18] Guo Q, Ban FX, Xia WQ, Shu YN, Liu YQ, Liu SS, Pan LL, Wang XW. The essential role of clathrin-mediated endocytosis and early endosomes in the trafficking of begomoviruses through the primary salivary glands of their whitefly vectors. J Virol. 2023;97:e0106723. 37855618 10.1128/jvi.01067-23 PMC10688308

[19] He YZ, Wang YM, Yin TY, Fiallo-Olivé E, Liu YQ, Hanley-Bowdoin L, Wang XW. A plant DNA virus replicates in the salivary glands of its insect vector via recruitment of host DNA synthesis machinery. Proc Natl Acad Sci U S A. 2020;117:16928–37. 32636269 10.1073/pnas.1820132117 PMC7382290

[20] Wang XR, Wang C, Ban FX, Ghanim M, Pan LL, Qian LX, Liu YQ, Wang XW, Liu SS. Apoptosis in a whitefly vector activated by a begomovirus enhances viral transmission. mSystems. 2020;5:e00433-20. 32963100 10.1128/mSystems.00433-20 PMC7511215

[21] Wang SF, Guo HJ, Zhu-Salzman K, Ge F, Sun YC. PEBP balances apoptosis and autophagy in whitefly upon arbovirus infection. Nat Commun. 2022;13:846. 35149691 10.1038/s41467-022-28500-8 PMC8837789

[22] Wang YM, He YZ, Ye XT, Guo T, Pan LL, Liu SS, Ng JCK, Wang XW. A balance between vector survival and virus transmission is achieved through JAK/STAT signaling inhibition by a plant virus. Proc Natl Acad Sci U S A. 2022;119:e2122099119. 36191206 10.1073/pnas.2122099119 PMC9564230

[23] Wang XR, Shao Y, Wang C, Liu YQ. Effects of heat stress on virus transmission and virus-mediated apoptosis in whitefly *Bemisia tabaci*. Arch Insect Biochem Physiol. 2021;110:e21857. 34859483 10.1002/arch.21857

[24] Ritossa F. A new puffing pattern induced by temperature shock and DNP in *Drosophila*. Experientia. 1962;18:571–3.

[25] Wojda I. Temperature stress and insect immunity. JTherm Biol. 2017;68:96–103. 28689727 10.1016/j.jtherbio.2016.12.002

[26] Gonzále-Tokman D, Córdoba-Aguilar A, Dáttilo W, Lira-Noriega A, Sánchez-Guillén RA, Villalobos F. Insect responses to heat: physiological mechanisms, evolution and ecological implications in a warming world. Biol Rev Camb Philos Soc. 2020;95:802–21. 32035015 10.1111/brv.12588

[27] King AM, Macrae TH. Insect heat shock proteins during stress and diapause. Annu Rev Entomol. 2015;60:59–75. 25341107 10.1146/annurev-ento-011613-162107

[28] Baler R, Dahl G, Voellmy R. Activation of human heat shock genes is accompanied by oligomerization, modification, and rapid translocation of heat shock transcription factor HSF1. Mol Cell Biol. 1993;13:2486–96. 8455624 10.1128/mcb.13.4.2486-2496.1993 PMC359569

[29] Sakurai H, Enoki Y. Novel aspects of heat shock factors: DNA recognition, chromatin modulation and gene expression. FEBS J. 2010;277:4140–9. 20945530 10.1111/j.1742-4658.2010.07829.x

[30] Bai J, Liu YC, Wei R, Wang YC, Gong WR, Du YZ. Knockdown of heat shock transcription factor 1 decreases temperature stress tolerance in *Bemisia tabaci* MED. Sci Rep. 2022;12:16059. 36163391 10.1038/s41598-022-19788-z PMC9512819

[31] Ma CS, Ma G, Pincebourde S. Survive a warming climate: insect responses to extreme high temperatures. Annu Rev Entomol. 2021;66:163–84. 32870704 10.1146/annurev-ento-041520-074454

[32] Liu SS, Colvin J, Barro PJD. Species concepts as applied to the whitefly *Bemisia tabaci* systematics: how many species are there? J Integr Agri. 2012;11:176–86.

[33] Tsai WA, Brosnan CA, Mitter N, Dietzgen RG. Perspectives on plant virus diseases in a climate change scenario of elevated temperatures. Stress Biol. 2022;2:37. 37676437 10.1007/s44154-022-00058-x PMC10442010

[34] Dash SP, Dipankar P, Burange PS, Rouse BT, Sarangi PP. Climate change: how it impacts the emergence, transmission, resistance and consequences of viral infections in animals and plants. Crit Rev Microbiol. 2021;47:307–22. 33570448 10.1080/1040841X.2021.1879006

[35] Akerfelt M, Morimoto RI, Sistonen L. Heat shock factors: integrators of cell stress, development and lifespan. Nat Rev Mol Cell Biol. 2010;11:545–55. 20628411 10.1038/nrm2938 PMC3402356

[36] Reyes A, Navarro AJ, Diethelm-Varela B, Kalergis AM, Gonzále PA. Is there a role for HSF1 in viral infections? FEBS Open Bio. 2022;12:1112–24. 35485710 10.1002/2211-5463.13419 PMC9157408

[37] Filone CM, Caballero IS, Dower K, Mendillo ML, Cowley GS, Santagata S, Rozelle DK, Yen J, Rubins KH, Hacohen N, et al. The master regulator of the cellular stress response (HSF1) is critical for orthopoxvirus infection. PLoS Pathog. 2014;10:e1003904. 24516381 10.1371/journal.ppat.1003904 PMC3916389

[38] Tsai TT, Chen CL, Tsai CC, Lin CF. Targeting heat shock factor 1 as an antiviral strategy against dengue virus replication *in vitro* and *in vivo*. Antiviral Res. 2017;145:44–53. 28733114 10.1016/j.antiviral.2017.07.008

[39] Rawat P, Mitra D. Cellular heat shock factor 1 positively regulates human immunodeficiency virus-1 gene expression and replication by two distinct pathways. Nucleic Acids Res. 2011;39:5879–92. 21459854 10.1093/nar/gkr198 PMC3152347

[40] Wang FW, Wu XR, Liu WJ, Liao YJ, Lin S, Zong YS, Zeng MS, Zeng YX, Mai SJ, Xie D. Heat shock factor 1 upregulates transcription of Epstein-Barr Virus nuclear antigen 1 by binding to a heat shock element within the BamHI-Q promoter. Virology. 2011;421:184–91. 22018489 10.1016/j.virol.2011.10.001

[41] Hunt D, Raivich G, Anderson PN. Activating transcription factor 3 and the nervous system. Front Mol Neurosci. 2012;5:7. 22347845 10.3389/fnmol.2012.00007 PMC3278981

[42] Liu S, Li Z, Lan S, Hao H, Baz AA, Yan X, Gao P, Chen S, Chu Y. The dual roles of activating transcription factor 3 (ATF3) in inflammation, apoptosis, ferroptosis, and pathogen infection responses. Int J Mol Sci. 2024;25:824. 38255898 10.3390/ijms25020824 PMC10815024

[43] Shu MF, Du T, Zhou G, Roizman B. Role of activating transcription factor 3 in the synthesis of latency-associated transcript and maintenance of herpes simplex virus 1 in latent state in ganglia. Proc Natl Acad Sci U S A. 2015;112:E5420–6. 26305977 10.1073/pnas.1515369112 PMC4593112

[44] Li XY, Pang LR, Chen YG, Weng SP, Yue HT, Zhang ZZ, Chen YH, He JG. Activating transcription factor 4 and X box binding protein 1 of *Litopenaeus vannamei* transcriptional regulated white spot syndrome virus genes *Wsv023* and *Wsv083*. PLoS One. 2013;8:e62603. 23638122 10.1371/journal.pone.0062603 PMC3634759

[45] Tue NT, Yoshioka Y, Mizoguchi M, Yoshida H, Zurita M, Yamaguchi M. DREF plays multiple roles during *Drosophila* development. Biochim Biophys Acta Gene Regul Mech. 2017;1860:705–12. 28363744 10.1016/j.bbagrm.2017.03.004

[46] Ito T, Sakai A, Maruyama M, Miyagawa Y, Okada T, Fukayama H, Suzuki H. Dorsal Root Ganglia Homeobox downregulation in primary sensory neurons contributes to neuropathic pain in rats. Mol Pain. 2020;16:1744806920904462. 32000573 10.1177/1744806920904462 PMC7099666

[47] Osterloh A, Breloer M. Heat shock proteins: linking danger and pathogen recognition. Med Microbiol Immunol. 2008;197:1–8. 17638015 10.1007/s00430-007-0055-0

[48] Wronska AK, Bogus MI. Heat shock proteins (HSP 90, 70, 60, and 27) in *Galleria mellonella* (Lepidoptera) hemolymph are affected by infection with *Conidiobolus coronatus* (Entomophthorales). PLoS One. 2020;15:e0228556. 32027696 10.1371/journal.pone.0228556 PMC7004346

[49] Wang XR, Wang C, Ban FX, Zhu DT, Liu SS, Wang XW. Genome-wide identification and characterization of HSP gene superfamily in whitefly (*Bemisia tabaci*) and expression profiling analysis under temperature stress. Insect Sci. 2019;26:44–57. 28714602 10.1111/1744-7917.12505

[50] Wu S, Zhao Y, Wang D, Chen Z. Mode of action of heat shock protein (HSP) inhibitors against viruses through host HSP and virus interactions. Genes. 2023;14:792. 37107550 10.3390/genes14040792 PMC10138296

[51] Gorovits R, Czosnek H. The involvement of heat shock proteins in the establishment of tomato yellow leaf curl virus infection. Front Plant Sci. 2017;8:355. 28360921 10.3389/fpls.2017.00355 PMC5352662

