# Supplementary material for: Heat stress promotes the accumulation of tomato yellow leaf curl virus in its insect vector by activating heat shock factor
Source: Crop Health. 2024 Dec 17;2(1):19. doi: 10.1007/s44297-024-00039-8 (PMC12825969; doi:10.1007/s44297-024-00039-8)
Supplement: Supplementary file 1 — Supplementary Material 1. [file 44297_2024_39_MOESM1_ESM.docx]

Supplementary Information for

**Heat stress promotes the accumulation of tomato yellow leaf curl virus in its insect vector by activating heat shock factor**

Yu-Meng Wang ^1,2^, Ya-Zhou He ^2^, Wilmer J. Cuellar ^3^, Xiao-Wei Wang ^1,^*

^1^ State Key Laboratory of Rice Biology and Breeding, Ministry of Agriculture Key Lab of Molecular Biology of Crop Pathogens and Insects, Key Laboratory of Biology of Crop Pathogens and Insects of Zhejiang Province, Institute of Insect Sciences, Zhejiang University, 310058 Hangzhou, China

^2^ College of Plant Protection, Nanjing Agricultural University, 210095 Nanjing, China

^3^ Virology Laboratory, Cassava Program, International Center for Tropical Agriculture (CIAT), Km 17 Recta Cali-Palmira 763537, Colombia

* Corresponding author

*E-mail address:* [xwwang@zju.edu.cn](mailto:xwwang@zju.edu.cn)

| Category or gene ID | Description | Fold change | Q value |
| --- | --- | --- | --- |
| *HSPs Response to heat* |  |  |  |
| LOC109039224 | heat shock protein 70-5 | 165.15 | 9.47E-74 |
| LOC109040186 | heat shock protein 70-11 | 152.88 | 1.46E-63 |
| LOC109040256 | heat shock protein 70-12 | 149.08 | 4.2E-74 |
| LOC109035112 | heat shock protein 70-2 | 92.30 | 1.7E-45 |
| LOC109038391 | heat shock protein 70-4 | 79.95 | 3.55E-40 |
| LOC109042138 | heat shock protein 70-13 | 38.97 | 2.90E-144 |
| LOC109031295 | heat shock protein 90A2 | 2.54 | 1.97E-26 |
| LOC109037043 | heat shock protein 19.5 | 25.61 | 6.26E-163 |
| LOC109042427 | heat shock protein 21.5 | 16.39 | 8.28E-87 |
| LOC109044373 | heat shock protein 19.4 | 74.48 | 1.59E-161 |
| LOC109031274 | protein lethal(2)essential for life-like | 13.19 | 1.94E-91 |
| LOC109042392 | dnaJ protein homolog 1-like | 2.32 | 4.5E-32 |
| *Transcription or protein folding* | |  |  |
| LOC109034327 | protein unc-45 homolog B | 4.07 | 2.73E-30 |
| LOC109043279 | activating transcription factor 3 | 2.37 | 0.0088 |
| LOC109039225 | DDB1- and CUL4-associated factor 8-like | 2.16 | 1.81E-33 |
| LOC109031372 | nocturnin isoform X3 | 2.12 | 7.87E-06 |
| LOC109040432 | zinc finger BED domain-containing protein 1-like | 0.48 | 0.0024 |
| LOC109039592 | neurogenic differentiation factor 6-A | 0.41 | 0.0037 |
| LOC109036558 | dorsal root ganglia homeobox protein-like | 0.25 | 0.0056 |

**Table S1 DEGs response to heat and involved in transcription or protein folding**

**Table S2 Primers used in this study**

| Primer name |  | Sequences (5'-3') | Length | Purpose |
| --- | --- | --- | --- | --- |
| COI | F | TTGATTTTTTGGTCATCCAGAAGT | 750bp | Identification of *Bemisia tabaci* cryptic species |
|  | R | TCCAATGCACTAATCTGCCATATTA |  |  |
| qTYLCV | F | GAAGCGACCAGGCGATATAA | 189 bp | qPCR for TYLCV total DNA |
|  | R | GGAACATCAGGGCTTCGATA |  |  |
| q-β-actin | F | TCTTCCAGCCATCCTTCTTG | 173 bp | q(RT)-PCR for *β-actin* |
|  | R | CGGTGATTTCCTTCTGCATT |  |  |
| qBtHSP70-4 | F | AGAGCCCTGCGAAGACTGCG | 178bp | qRT-PCR for *BtHSP70-4* |
|  | R | CTGGGTGGAGGGTTGAGCGG |  |  |
| qBtHSP70-5 | F | TGTGTCGGTGTGTGGCAGCA | 181bp | qRT-PCR for *BtHSP70-5* |
|  | R | TCCGTCCGATGAGGCGCTTG |  |  |
| qBtHSP70-11 | F | GCCCTGGCATACGGCTTGGA | 238bp | qRT-PCR for *BtHSP70-11* |
|  | R | GAGGGTTGGTGCGGAGGTCC |  |  |
| qBtHSP70-12 | F | ACGCGCCAAGATAGCTGCCA | 170bp | qRT-PCR for *BtHSP70-12* |
|  | R | CCTCCACATCGGCGAGGGTG |  |  |
| qBtHSP70-13 | F | GCAGGCGGTAGAGGAGGCAG | 198bp | qRT-PCR for *BtHSP70-13* |
|  | R | CCAGCTCCACCACCTGCTCC |  |  |
| qBtHSP19.4 | F | ACCCGCCGTTACCTTCTGCC | 158bp | qRT-PCR for *BtHSP19.4* |
|  | R | GCTGGGGCGTTGGTTTTGGT |  |  |
| qBtHSP19.5 | F | CTCACGGCGATCAGTGCGGA | 158bp | qRT-PCR for *BtHSP19.5* |
|  | R | GGGTGATGAACTGGATGGCCGA |  |  |
| dsRNA-GFP | F | TAATACGACTCACTATAGGGCTCGTGACCACCCTGACCTAC | 247 bp | *GFP* dsRNA synthesis |
|  | R | TAATACGACTCACTATAGGGGTTCACCTTGATGCCGTTCTT |  |  |
| dsRNA-BtHSF | F | TAATACGACTCACTATAGGGGTGTCTGTTACTGGTTCTCT | 400 bp | *BtHSF* dsRNA synthesis |
|  | R | TAATACGACTCACTATAGGGATGACCAGACCTGTTGAAAG |  |  |
| qBtHSF | F | AACAGCGGCAGTTCAGATTC | 143 bp | qRT-PCR for *BtHSF* |
|  | R | GTGATGGTGGGTGACAATGG |  |  |
| pAc5.1-BtHSF | F | GGGGTACCTATGCACCCAATTACGGAGGT | 2283 bp | Expression of BtHSF in S2 cells (KpnI and XbaI sites are underlined) |
|  | R | GCTCTAGACTTTTTCTTCTTCTTTTCGCACTGG |  |  |
| TYLCV-P M1 | F | TTTCTCTATCGATAGGTACCTTTATTGATTTGATCTTTTGATTTTG | 158bp | Construction of pGL3-basic reporter vector |
| TYLCV-P M2 | F | TTTCTCTATCGATAGGTACCTTTATTGATTTGAGCAATTGCTTTTG | 158bp | Construction of pGL3-basic reporter vector |
| TYLCV-P-WT | F | TTTCTCTATCGATAGGTACCTTTATTGATTTGATTTTTGAATTTT | 158bp | Construction of pGL3-basic reporter vector |
|  | R | AGTACCGGAATGCCAAGCTTGTTGAAATGAATTGG |  |  |


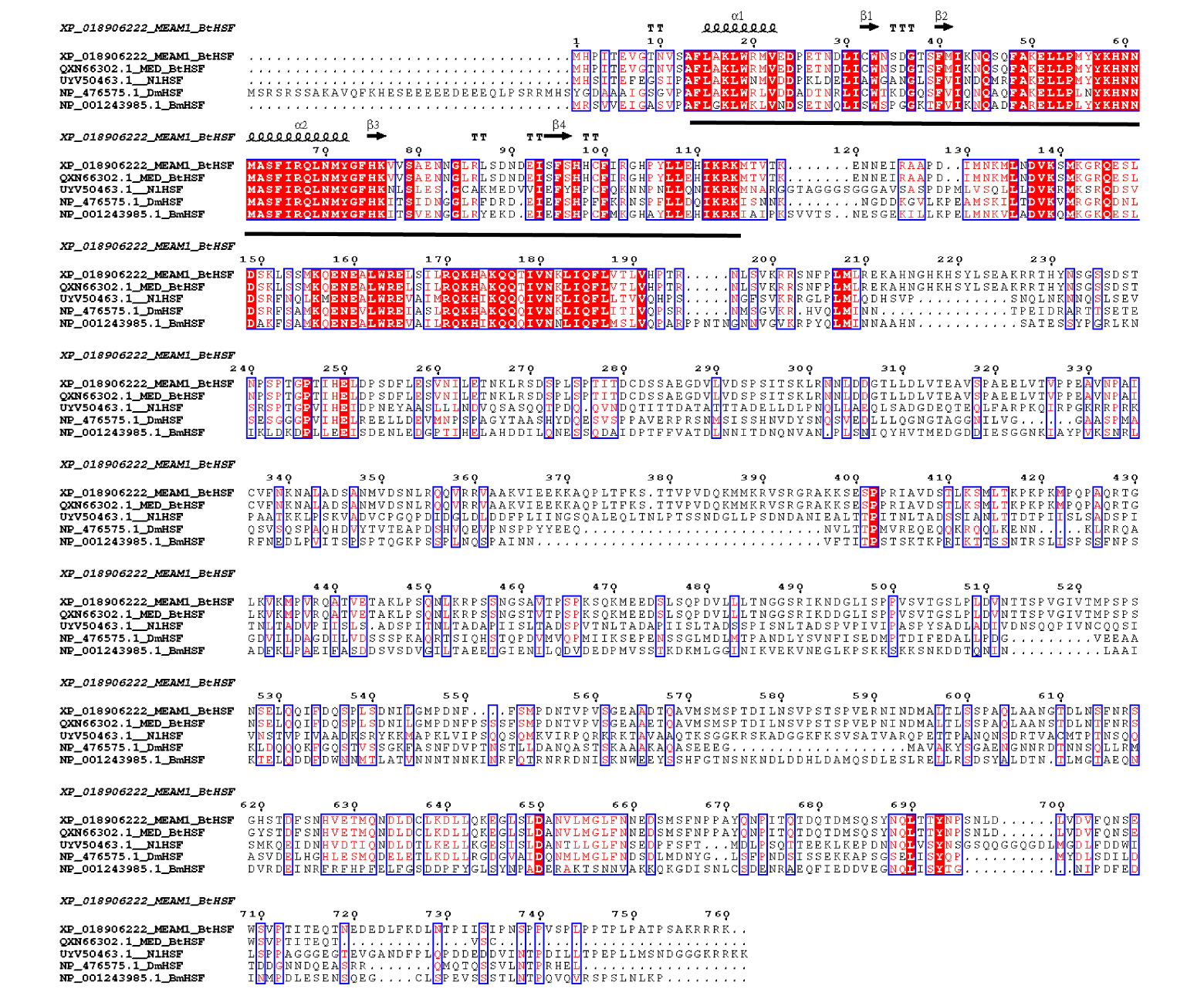


**Fig S1. Multiple** **sequence alignment of HSF from various insect species and secondary structure of BtHSF.** Alignment of the HSF deduced amino acid sequences of MEAM1 *Bemisia tabaci* (XP_018906222), MED *B. tabaci* (QXN66302.), *Nilaparvata lugens* (UYV50463.1), *Drosophila melanogaster* (NP_476575.1), *Bombyx mori* (NP_001243985.1). The DNA-binding (DBD) motif are underlined in black. Sequence alignment and its visualization were performed by MEGA 7.0 and ESPript 3.0 (https://espript.ibcp.fr/ESPript/cgi-bin/ESPript.cgi). Protein secondary structure prediction was performed by SWISS-MODEL (https://swissmodel.expasy.org/).


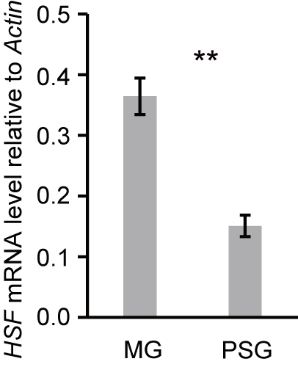


**Fig S2. Relative transcript levels of *BtHSF* in midgut (MG) and primary salivary glands (PSG) of non-viruliferous whiteflies.** Data are presented as mean ± SEM of three independent experiments with 100 midguts, 100 primary salivary glands in each replicate and analyzed statistically using independent-samples t-test (**, *p* < 0.01).


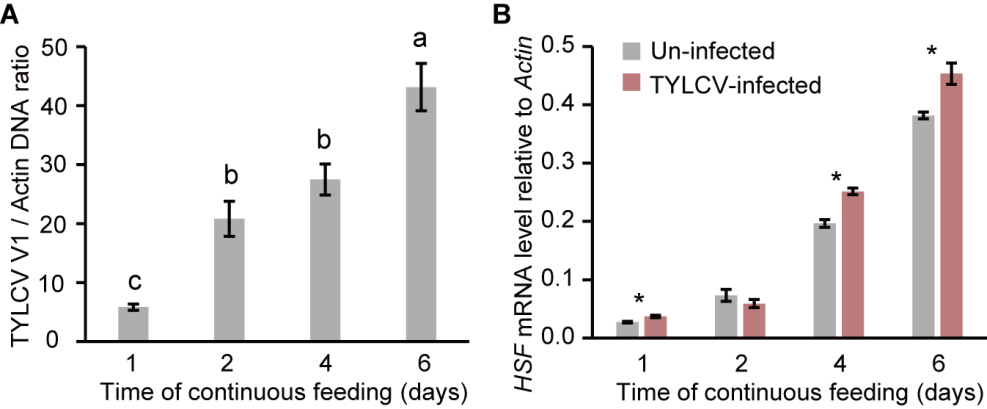


**Fig S3. Effect of TYLCV infection on *BtHSF* expression in whiteflies.** Quantitative analysis of TYLCV DNA (A) and *BtHSF* mRNA level (B) in whiteflies at 1, 2 ,4 and 6 days during continuously feeding on TYLCV infected tomato plants. Data are presented as mean ± SEM of three independent experiments and analyzed statistically using one-way ANOVA, LSD test (*p* < 0.05) in (A) and independent-samples t-test ( *, *p* < 0.05; **, *p* < 0.01) in (B).
